# Oral Health-Related Knowledge, Attitudes and Behaviours of Elementary School Teachers

**DOI:** 10.3390/ijerph18116028

**Published:** 2021-06-03

**Authors:** Gülçin Yılmaz, Abanoub Riad, Martin Krsek, Hanefı Kurt, Sameh Attia

**Affiliations:** 1 Department of Prosthodontics, Faculty of Dentistry, Istanbul Medipol University, Istanbul 34810, Turkey; hkurt@medipol.edu.tr; 2 Department of Public Health, Faculty of Medicine, Masaryk University, 62500 Brno, Czech Republic; abanoub.riad@med.muni.cz (A.R.); krsek@med.muni.cz (M.K.); 3 Department of Oral and Maxillofacial Surgery, Justus-Liebig-University, Klinikstrasse 33, 35392 Giessen, Germany

**Keywords:** attitude to health, behavior, oral health, oral hygiene, schoolteachers, turkey, wounds and injuries

## Abstract

Background: elementary schoolteachers play a central role in shaping their students’ beliefs, attitudes, and behaviours related to health and oral hygiene. This study was designed to evaluate Turkish schoolteachers’ levels of oral health knowledge, attitudes, and behaviours. Methods: A cross-sectional survey-based study was conducted among elementary schoolteachers in Istanbul using a validated self-administered questionnaire. The questionnaire was composed of 36 multiple-choice items categorised into six sections, and the participants were recruited using convenience sampling. (3) Results: A total of 385 elementary schoolteachers participated in this study. The majority were female (62.2%), qualified with a licensure degree (81.3%) and working in public schools (86.5%). Female gender and greater work experience were found to be promoters for oral health knowledge and positive attitudes. The correlation between their perceived knowledge and actual knowledge was very weak, thus suggesting that the teachers are inclined to overestimate their knowledge. Conclusions: The Turkish elementary schoolteachers showed satisfactory oral health knowledge and attitudes toward oral health education. The teachers’ knowledge about dental trauma management was inadequate, necessitating urgent educational interventions, especially for physical education teachers, who are at the greatest risk of encountering such events during their work. The oral hygiene behaviours were not associated with teachers’ oral health knowledge, attitudes, or practice, thus requiring further investigation.

## 1. Introduction

Oral hygiene measures in childhood lead to healthy teeth and oral mucosa, providing optimal general health conditions [1]. Oral diseases are depicted as a major public health challenge, especially in school children. A total of 90% of them are suffering from dental caries, with increasing incidences in Asian and Latin American countries as reported by the Global Burden of Disease (GBD) in its 2005 report [2]. The prevalence of tooth decay in school children ranged between 60 and 90%. The incidence of caries is rising rapidly in developing countries [2].

It had been reported by the United Nations Educational, Scientific and Cultural Organization (UNESCO) that the number of teachers at the primary school level worldwide by the year 1993 was around 23.9 million [3]. Schools continue to be an essential environment, offering an ideal and effective method to manage over 1 billion children worldwide [4]. Preschools and primary schools have great potential to influence child’s health behaviour [5,6]. Children spend a significant amount of time at school, especially at the age when their habits are shaped. Therefore, the role of teachers is critical in these developmental stages of the child [7].

The education of school-age children in oral health is crucial because healthy oral habits occur at a young age. The importance of teaching children (infants, preschoolers, or schoolchildren) about oral hygiene was recognised as early as 1878 [8,9]. Schools are an optimal location for providing oral health education, as these services can be given similarly and widely to all children, especially those who do not have access to other health resources and cannot receive professional dental care [10,11].

One of the critical issues in oral health is the treatment of dental injuries [12]. Children participate in sports activities at school, and in cases of close contact or physical activity, injuries may occur due to reasons such as falls or accidents [13]. In these trauma cases, successful management of the process from the moment of the event to the visit to the dentist significantly increases the chances of successful post-trauma treatments [14]. The good management of this process depends on the teacher’s level of knowledge. Correct guidance of the child and their parents can give the dentist a chance for early intervention. A teacher needs to know what to do in an emergency regarding primary and permanent teeth [15]. Dental trauma in industrialised countries ranges from 16 to 40% for six-year-olds and 4 to 33% for 12- to 14-year-olds; in some Latin American countries, it is about 15% of schoolchildren; in the Middle East, it is about 5 to 12% among 6- to 12-year-olds [16]. Many studies have shown that getting support from teachers is successful in improving the oral health of school children [17].

However, according to some reports, teachers were hesitant to participate in oral health programs that require supervision [18]. The reason for this is thought to be due to the teachers’ lack of knowledge on oral health [19]. Since schoolteachers are models for school students, their oral health knowledge must be good, and their oral health behaviours and attitudes must follow professional advice [1]. For this reason, their knowledge and attitudes about oral health are important both for their oral health and for the children they influence and teach as a model [6]. Oral health is an integral part of overall health and well-being. Individuals with a healthy mouth live without pain, discomfort, and embarrassment while talking, eating, and socialising [20,21]. One study showed that school-age adolescents suffering from poor oral health were 12 times more often deprived of activities compared to their peers [22]. More than 50 million school classes are lost worldwide due to poor oral health. This can affect the student’s school performance and subsequent success in his life [23]. Ideally, primary schoolteachers can give information about good oral health, dental and gum care, proper oral hygiene, use of fluoride, proper dietary advice, and the benefit of routine dental visits [24]. The goal of oral health education is to improve knowledge within the target population, leading them to be individuals with better oral health and to adopt positive oral health behaviours [16].

Recent studies revealed that oral-health promotion programs have a significant effect in improving dental health status and reducing the cost of healthcare systems. Therefore, oral-health promotion programs revealed their effects on children’s oral health and on parental dental treatment expenses [25]. Education in and through the media had an increasing tendency that can be seen in most health-promoting media literacy classes being taught in schools [26]. In order to prevent the spread of infectious diseases, health personnel are advised to use audiovisual media to educate the population about health [27]. Mass media campaigns tend to be helpful in influencing beliefs of the general public and healthcare practitioners by increasing the alignment between beliefs and current evidence and promoting self-management concepts [28].

## 2. Materials and Methods

### 2.1. Design

This study was conducted in Istanbul, Turkey, between July and September 2020 as an analytical cross-sectional survey-based study using an online self-administered questionnaire (SAQ) of multiple-choice items in order to evaluate the oral health self-perceived knowledge, actual knowledge, attitudes, behaviours, and practice of elementary school teachers. The questionnaire was electronically developed using SurveyMonkey (SVMK Inc. San Mateo, CA, USA 2020), and the target subjects received a uniform resource locator (URL) and a quick response (QR) code from their school administrator/principal to take part in the study.

### 2.2. Participants

According to the official data of the Istanbul governorship, as of May 2019, there were 3,175,285 students enrolled in 7437 schools (3790 private and 3647 publicly funded) with 168,527 teachers. The adequate sample size for this study was calculated using Epi InfoTM version 7.2.4 (CDC. Atlanta, GA, USA 2020), which indicated that a total of 384 teachers was required in order to achieve conclusive results with a confidence level of 95% and an error margin of 5%. Participation in this study was entirely voluntary and not financially compensated or incentivised by other means to minimise self-selection bias. The participants were able to withdraw from the study at any time without the need to explain the reason.

### 2.3. Instrument

The items of the SAQ used in this study were adopted from previous studies with relevant purposes, including the assessment of schoolteachers’ oral health knowledge and attitudes, assessment of schoolteachers’ knowledge and practice regarding dental injuries, and evaluation of oral hygiene behaviours [1,2,5,6,7]. The instrument’s content validity was evaluated by a panel of experts in dental public health who reviewed the proposed items in terms of clarity and relevance.

The SAQ had 36 multiple-choice items categorised into six sections. Section I (five items) was about demographic data such as gender, length of work experience, education status and branch. Section II (four items) was about perceived oral health knowledge assessed by a five-point Likert scale ranging from totally disagree to totally agree. Section III (10 items) was about actual oral health knowledge, which was designed to correlate with the items of Section II. Section IV (four items) was about the attitudes towards oral health education assessed by a five-point Likert scale ranging from totally disagree to totally agree. Section V (five items) was adapted from Hiroshima University Dental Behavioral Inventory (HU-DBI) aiming to evaluate oral health behaviours [29]. Section VI (eight items) was about the teachers’ experience and practice of oral health education (Supplementary Materials).

### 2.4. Ethics

This study was conducted following the Declaration of Helsinki for the ethical principles of medical research involving human subjects. It was reported according to the Strengthening the Reporting of Observational Studies in Epidemiology (STROBE) statement for cross-sectional studies [30,31]. Ethical approval was granted by the institutional review board (IRB) of Istanbul Medipol University under the code of 10840098-772.02-E.34168 on 8 July 2020. Written permission was obtained from each participating school’s principal/administrator to carry out the survey; electronic informed consent was required from each invited teacher before filling in the questionnaire.

### 2.5. Statistics

All statistical analyses were performed using the Statistical Package for the Social Sciences (SPSS) version 27.0 (SPSS Inc., Chicago, IL, USA, 2020). Descriptive statistics were carried out primarily for the normality of data, followed by the frequencies, percentages and central tendencies of demographic variables, oral health-related knowledge, attitudes, behaviours, and practice.

For the total score of actual knowledge, each question had one point for the correct answer and zero points for wrong answers. The total score of perceived knowledge and the total score of attitudes were calculated by pooling the scores of each question in these sections that were originally rated by a five-point Likert scale. For the total score of oral hygiene behaviors, one point was given for the favorable behavior and zero points were given to the unfavorable behavior.

Consequently, inferential statistics were carried out using the Chi-squared test (*χ*^2^), Mann–Whitney test (*U*), and Kruskal–Wallis (*H*) test to evaluate the impact of demographic variables on teachers’ oral health knowledge, attitudes, and behaviours. Spearman’s correlation test (*ρ*) was performed to evaluate the association between actual knowledge domains and perceived knowledge items. The strengths of correlation are described by the value of (*ρ*): 0.01 to 0.39 “weak”; 0.40 to 0.69 “moderate”; 0.70 to 0.99 “strong”; 1.0 “perfect” [32]. All inferential tests were carried out with a confidence level of 95% and significance value *p* ≤ 0.05.

## 3. Results

### 3.1. Demographic Characteristics

A total of 385 elementary schoolteachers from Istanbul, Turkey, participated in this study. The majority were females (62.2%), qualified with a licensure degree (81.3%) and working in public schools (86.5%). While 87 (22.6%) participants had one to five years of work experience, 111 (28.8%) have worked for 6 to 10 years, 89 (23.1%) had worked for 11 to 20 years, and 98 (25.5%) had worked for over 20 years. Eleven (2.9%) participants were teachers of physical education (Table 1).

### 3.2. Oral Health-Related Knowledge

Oral health-related knowledge of teachers was evaluated in two main constructs—self-perceived knowledge and actual knowledge. Perceived knowledge is one’s self-assessment or feeling of knowing the information, and it should be distinguished from and assessed concurrently with actual knowledge in order to find out any knowledge discrepancies that indicate “knowledge illusion” [33,34,35]. The four domains of knowledge tested in this study were (i) primary dentition, (ii) oral diseases risk factors, (iii) oral hygiene methods, and (iv) dental trauma management.

Regarding their perceived knowledge, 76.6%, 64.2%, 82.3%, and 36.9% of the participants agreed that they have sufficient knowledge about primary dentition, oral diseases risk factors, oral hygiene methods, and dental trauma management, respectively. The teachers with >10 years of work experience had higher levels of perceived knowledge in all four knowledge domains (*U* = 15,919, 14,819.5, 17,994.5, and 14,663.5; *p =* 0.01, <0.001, 0.589, and <0.001, respectively) than their colleagues with ≤10 years of experience. Female teachers had higher levels of all four perceived knowledge domains (U 13,690, 15,446, 14,602, and 16,976; *p* < 0.001, 0.054, 0.003, and 0.706, respectively) than their male colleagues. In general, the teachers at private schools had higher perceived knowledge levels than the teachers at public schools.

Regarding their actual knowledge, the participants had to answer 10 multiple choice questions with one correct answer (two items: primary dentition; four items: oral diseases risk factors; two items: oral hygiene methods; two items: dental trauma management). All items of the second (oral diseases risk factors) and third domain (oral hygiene methods) were correctly answered by more than 50% of the participants. The two items of the fourth domain (dental trauma management) received the least number of correct answers 26% and 32.5%, respectively. The teachers with ≤10 years of experience had a significantly higher level of actual knowledge in the first domain (*U* = 16,446; *p =* 0.043) and a lower level in the fourth domain (*U* = 14,924; *p =* 0.007) than their colleagues with >10 years of experience. Female teachers had a significantly higher level of actual knowledge in the second domain (*U* = 13,648.5; *p* < 0.001) than their male colleagues. The difference between private schoolteachers and public schoolteachers in actual knowledge domains was not statistically significant (Table 2).

### 3.3. Oral Hygiene Behaviours

Oral hygiene behaviours were evaluated using five items adapted from the Turkish version of HU-DBI (items no. 1, 4, 9, 12, and 16) [29,36]. While 243 (63.1%) participants reported that they do not worry about visiting the dentist, only 29 (7.5%) and 50 (13%) of them had used disclosing dye and a child-sized toothbrush. The teachers with ≤10 years of experience had a lower level of calculus detection (*χ*^2^ = 9.87; *p =* 0.002) compared to their colleagues with >10 years of experience. For the total behaviours score (0–5), no significant difference was found between participants in terms of gender, work experience, or school type (Table 3).

### 3.4. Oral Health Education Attitudes

The schoolteachers’ attitudes towards providing oral health education to their students were evaluated by four items assessed by a five-point Likert scale ranging from “strongly agree = 5 “to “strongly disagree = 1”. A total of 311 (80.8%) participants agreed that teachers should have a role in the oral health education of schoolchildren. The teachers with ≤10 years of experience (74.3%) had a significantly (*χ*^2^ = 11.22; *p* < 0.001) lower level of acceptance for their proposed role in providing oral health education compared to their colleagues with >10 years of experience (87.7%). A total of 294 (76.4%) participants agreed that teachers should receive oral health training as a part of general health training. The teachers with ≤10 years of experience (70.7%) had a significantly (*χ*^2^ = 7.23; *p =* 0.007) lower level of willingness for receiving oral health training compared to their colleagues with >10 years of experience (82.4%).

A total of 334 (86.8%) participants believed that oral health could affect the psychology of schoolchildren. The teachers with ≤10 years of experience (89.4%) had an insignificantly (*χ*^2^ = 3.01; *p =* 0.083) lower level of belief of oral health impact on schoolchildren’s psychology compared to their colleagues with >10 years of experience (89.8%). A total of 351 (91.2%) participants agreed that oral health education could benefit their schoolchildren. The teachers with ≤10 years of experience (83.8%) had an insignificantly (*χ*^2^ = 1.60; *p =* 0.207) lower level of belief of oral health benefits for their schoolchildren compared to their colleagues with >10 years of experience (93%). There was no significant difference between participants in any of the attitude items according to gender (female vs. male) or type of school (public vs. private) (Table 4).

### 3.5. Oral Health Education Experience

Only 67 (17.4%) participants reported receiving training on how to provide education in general hygiene-related topics, with a significant (*χ*^2^ = 5.48; *p =* 0.019) difference between private schoolteachers (28.8%) and public schoolteachers (15.6%), and a significant (*χ*^2^ = 7.91; *p =* 0.005) difference between teachers with >10 years of experience (23%) and ≤10 years of experience (12.1%). An even smaller fraction of participants (9.4%) reported receiving training on how to provide education in oral hygiene-related topics, without a significant difference according to the length of work experience (*χ*^2^ = 2.5; *p =* 0.114), gender (*χ*^2^ = 0.31; *p =* 0.579), or school type (*χ*^2^ = 0.34; *p =* 0.56).

While 51.7% of participants reported supervising the brushing habit of their schoolchildren, only 7.5% reported elevating the upper lip of their schoolchildren to check their teeth. Regarding their experience with teaching their own schoolchildren about oral health, 110 (28.6%) reported attempting to provide education on topics related to teeth and oral health, without a significant difference in terms of work experience (*χ*^2^ = 1.58; *p =* 0.209), gender (*χ*^2^ = 0.93; *p =* 0.334), and school type (*χ*^2^ = 0.5; *p =* 0.479) (Table 5).

**Figure 1:**
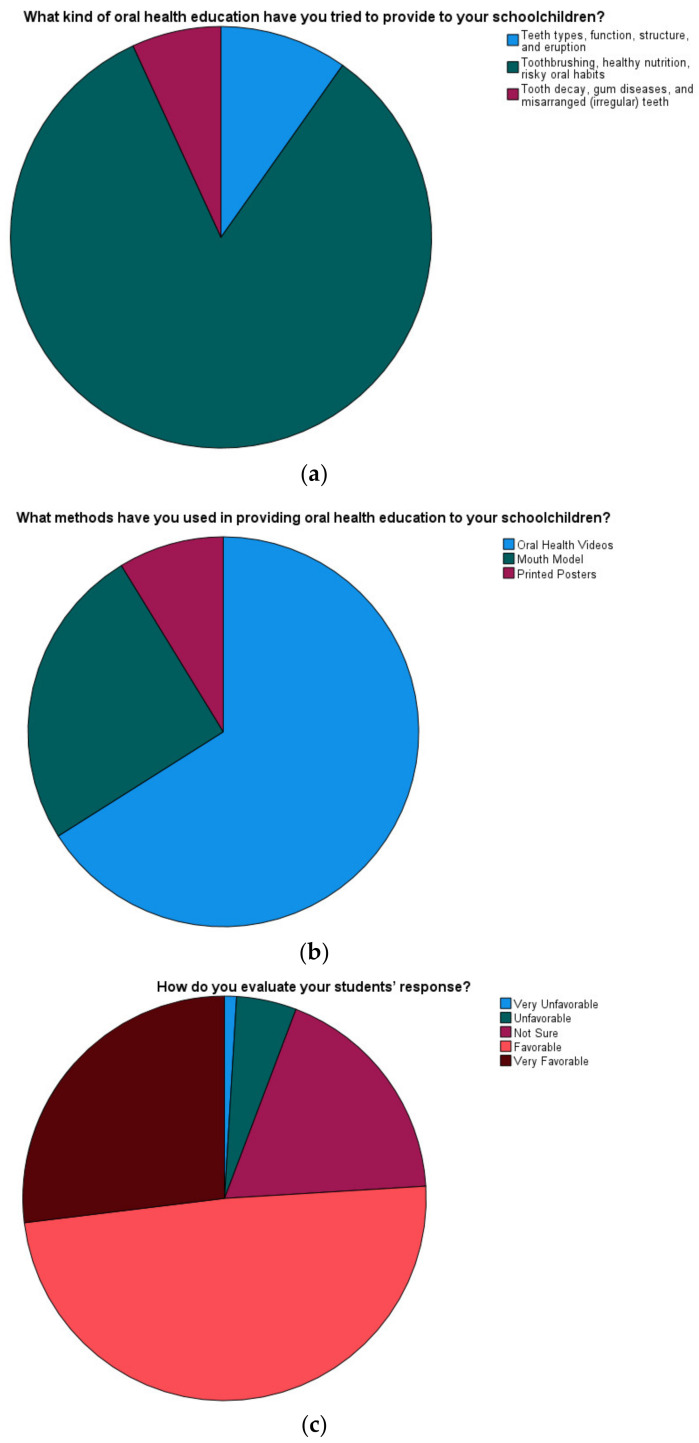
(**a**) Topics of oral health education provided to schoolchildren; (**b**) methods used in providing oral health education to schoolchildren; (**c**) schoolchildren’s feedback to oral health education provided by their teachers.

The most common topic for provided oral health education was oral hygiene (83.3%), followed by dental anatomy (9.8%) and oral disease (6.9%). While oral health videos (66%) were the most frequently used method in providing oral health education to schoolchildren, printed posters (8.7%) were the least common method. Regarding students’ feedback on the oral health education provided by their teachers, 75.9% had very favourable or favourable feedback, while only 5.8% had unfavourable or very unfavourable feedback (Figure 1).

### 3.6. Social Determinants of Oral Health Knowledge, Attitudes, and Behaviours

Female teachers had significantly higher levels of perceived oral health knowledge (*U* = 14,266.5; *p =* 0.003) and actual knowledge (*U* = 14,713.5; *p =* 0.011) than their male colleagues. Similarly, female teachers had insignificantly higher oral hygiene behaviours (*U* = 15,983.5; *p =* 0.175) than their male colleagues. Contrarily, male teachers had a higher level of attitudes (*U* = 17,099.5; *p =* 0.861) towards oral health education than female colleagues slightly.

The teachers with >10 years of work experience had higher levels of perceived oral health knowledge (*U* = 14,864.5; *p =* 0.001), actual knowledge (*U* = 18,027.5; *p =* 0.651), oral hygiene behaviours (*U* = 18,166.5; *p =* 0.739), and oral health education attitudes (*U* = 15,917; *p =* 0.019) than their colleagues with ≤10 years of experience.

The teachers with a master’s degree had slightly higher levels of perceived oral health knowledge (*U* = 10,101; *p =* 0.776), actual knowledge (*U* = 9213; *p =* 0.161), and oral health education attitudes (*U* = 9190; *p =* 0.162) than their colleagues with a licensure qualification. The only significant difference between teachers with a master’s degree and teachers with a licensure qualification was only found in terms of oral hygiene behaviours (*U* = 8024; *p =* 0.003).

The teachers at public schools had insignificantly higher levels of actual oral health knowledge (*U* = 8212; *p =* 0.544) and oral hygiene behaviours (*U* = 7787.5; *p =* 0.222), while the teachers at private schools had insignificantly higher levels of perceived oral health knowledge (*U* = 7910; *p =* 0.312) and oral health education attitudes (*U* = 8513.5; *p =* 0.871).

The physical education teachers had a significantly lower level of perceived oral health knowledge (*U* = 1260; *p =* 0.027) and actual knowledge (*U* = 1059; *p =* 0.005), and they had an insignificantly lower level oral health education attitudes (*U* = 2038; *p =* 0.956) and oral hygiene behaviours (*U* = 1321; *p =* 0.107). The physical education teachers were also the least knowledgeable in all actual knowledge domains, including dental trauma management (Table 6).

### 3.7. Correlation between Perceived Knowledge and Actual Knowledge

The overall score of perceived oral health knowledge was weakly correlated with the overall score of actual oral health knowledge (*ρ* = 0.265; *p* < 0.001). The perceived knowledge and the actual knowledge were positively correlated in all domains, even though the relationships between both knowledge constructs were either negligible or weak (0.008 to 0.274).

Both knowledge constructs were insignificantly (*ρ* = 0.080 and 0.071; *p =* 0.117 and 0.164) correlated in the first (primary dentition) and third domain (oral hygiene methods), respectively, while they were significantly (*ρ* = 0.208 and 0.274; *p* < 0.001 and < 0.001) correlated in the second (oral diseases risk factors) and fourth domain (dental trauma management), respectively.

While actual knowledge of the third domain (oral hygiene methods) was not significantly correlated with any perceived knowledge domains, the actual knowledge of the fourth domain (dental trauma management) was significantly correlated with all perceived knowledge domains (Table 7).

### 3.8. Association of Oral Health Knowledge and Behaviours

In all oral hygiene behaviour items of HU-DBI, the level of actual knowledge was higher in the teachers with favourable hygiene outcomes, except for the item of using disclosing agents, where the actual knowledge level was equal between the teachers who used disclosing agents and those who do not use them. Similarly, the level of perceived knowledge was higher in the teachers with favourable hygiene outcomes. On the other hand, the teachers who noticed white stick deposits had a significantly (*U* = 15,517.5; *p =* 0.019) higher perceived knowledge level. The overall behavior score (0–5) was weakly correlated with the overall score of perceived knowledge (*ρ* = 0.138; *p =* 0.007) and actual knowledge (*ρ* = 0.146; *p =* 0.004) (Table 8).

### 3.9. Association of Oral Health Knowledge and Attitudes

The teachers with higher levels of perceived knowledge were significantly more in favour of the teachers’ role in oral health education (*U* = 2983.5; *p =* 0.002), in agreement that teachers should receive oral health training as a part of general health training (*U* = 3553.5; *p =* 0.001), in agreement that oral health can affect the psychology of schoolchildren (*U* = 2154; *p =* 0.041), and in agreement that oral health education can benefit their schoolchildren (*U* = 699.5; *p =* 0.001).

While the teachers with higher levels of actual knowledge were significantly more in agreement that teachers should receive oral health training as a part of general health training (*U* = 4016.5; *p =* 0.016), they were insignificantly more in favour of the teachers’ role in oral health education (*U* = 3565; *p =* 0.058), and in agreement that oral health education can benefit their schoolchildren (*U* = 1399.5; *p =* 0.267) (Table 9).

### 3.10. Determinants of Oral Health Education Experience

The teachers who received education about general hygiene had significantly higher levels of perceived oral health knowledge (*U* = 5862; *p* < 0.001), actual knowledge (*U* = 8376.5; *p =* 0.005), and attitudes towards oral health education (*U* = 7591; *p* < 0.001). Similarly, the teachers who received education about oral hygiene had significantly higher levels of perceived oral health knowledge (*U* = 4161.5; *p =* 0.001), actual knowledge (*U* = 4628; *p =* 0.008), and attitudes towards oral health education (*U* = 3938; *p* < 0.001).

The teachers with higher levels of perceived oral health knowledge were more used to checking their students’ teeth (*U* = 4006; *p =* 0.043), supervising children’s brushing (*U* = 17751.5; *p =* 0.485), and teaching their students about oral health (*U* = 12,153; *p =* 0.002). Similarly, the teachers with higher levels of actual oral health knowledge were more used to checking their students’ teeth (*U* = 4513; *p =* 0.252), supervising children’s brushing (*U* = 13246.5; *p* < 0.001), and teaching their students about oral health (*U* = 12,129; *p =* 0.002).

The more positive attitudes towards oral health education, the higher the frequency of teachers had checking their students’ teeth (*U* = 3574; *p =* 0.005), supervising children’s brushing (*U* = 15,479; *p =* 0.006) and teaching their students about oral health (*U* = 11,720; *p =* 0.001) (Table 10).

## 4. Discussion

The primary objective of this study was to evaluate the elementary schoolteachers’ level of oral health knowledge, oral hygiene behaviours, and attitudes towards oral health education. The secondary objectives were to estimate the correlation between teachers’ perceived knowledge and actual knowledge, explore the discrepancies of teachers’ oral health knowledge, and evaluate the impact of teachers’ knowledge on their behaviours, attitudes, and practice.

Within the limits of our study, schoolteachers’ overall oral health knowledge was fair, except for dental trauma management. The correlation between perceived knowledge and actual knowledge was mainly weak in all the investigated domains. Our teachers’ perceived knowledge was significantly associated with their attitudes towards oral health education. The higher knowledge levels and the more positive attitudes were significant predictors for teachers’ actual engagement with providing oral health education to their children.

Across gender, the female teachers in our sample had significantly higher levels of perceived oral health knowledge (*U* = 14,266.5; *p =* 0.003) and actual oral health knowledge (*U* = 14,713.5; *p =* 0.011) than their male colleagues. The previous studies of elementary schoolteachers’ oral health knowledge in Saudi Arabia, India, Tanzania, and Uganda found that female teachers were more knowledgeable than their male colleagues [6,37,38,39,40,41,42,43]. While the difference between female and male teachers in terms of attitudes towards oral health education was statistically insignificant (*U* = 17,099.5; *p =* 0.861), the male teachers had slightly higher levels of positive attitudes, including the willingness to receive oral health education. Similarly, in Madinah (Saudi Arabia) and Rungwe (Tanzania), male teachers had more positive attitudes towards oral health [6,42,43]. The lower levels of males’ perceived knowledge can be utilised to explain their higher levels of positive attitudes towards oral health education. Contrarily, in Upper Galilee (Israel) and Al-Kharj (Saudi Arabia), female teachers had significantly higher levels of both oral health knowledge and attitudes [7,39]. Al-Jundi et al., 2005 suggested that the female teachers’ positive attitudes could be related to their caring nature and closeness to children [44].

The private schoolteachers had slightly higher levels of perceived oral health knowledge (*U* = 7910; *p =* 0.312) and lower levels of actual knowledge (*U* = 8212; *p =* 0.544) compared to the public schoolteachers. Vanka et al., 2012 found that the private schoolteachers in Bhopal (India) had a significantly lower level of oral health knowledge (*χ*^2^ = 15.421; *p =* 0.05) than the public schoolteachers [45]. However, in our sample, private schoolteachers (28.8%) reported more frequently that they received training in general hygiene than the public schoolteachers (15.6%); there was no significant difference in terms of receiving training about oral hygiene. Haloi et al., 2014 found that 82.2% of private schoolteachers had postgraduate degrees (PGs), while 65.2% of public schoolteachers held PGs in Mathura (India); this significant difference was found in our sample as 30.8% of private schoolteachers hold PGs while only 15% of the public schoolteachers do [46]. Therefore, the improved oral health outcomes of private school children in some developing countries should be fairly attributed to the fact that these children come from families with higher socioeconomic capacities that enable them to cover the tuition fees of private schools [47].

The length of work experience was a significant promoter for the teachers’ perceived oral health knowledge (*U* = 14864.5; *p =* 0.001) and their positive attitudes towards oral health education (*U* = 15,917; *p =* 0.019). The teachers with >10 years of work experience had significantly higher levels of both perceived knowledge (*U* = 14,663.5; *p* < 0.001) and actual knowledge (*U* = 15,924; *p =* 0.007) about what to do in case of dental trauma than their colleagues with ≤10 years of experience. This result is consistent with the findings of Junges et al., 2015 where the elementary schoolteachers with more than 15 years of work experience were more likely to know the appropriate conduct for handling teeth avulsion and crown fracture situations, thus suggesting that greater work experience could provide opportunities for schoolteachers to witness dental trauma events at schools or home [48]. The same conclusion had been previously drawn from cross-sectional studies in Singapore and Brazil [49,50].

On analysing the relationship between knowledge constructs, the schoolteachers’ perceived knowledge and actual knowledge were positively correlated in all the investigated domains, thus ruling out any critical knowledge discrepancies. Given the low correlation coefficients found in our sample, the schoolteachers’ perceived oral health knowledge can weakly predict their actual knowledge; therefore, it should not be exclusively used to evaluate the teachers’ knowledge in future studies. While the mean score of perceived knowledge was 14.91 ± 2.68 (total 20 points), the mean score of actual knowledge was 5.83 ± 1.73 (total 10 points), thus suggesting that the perceived knowledge score (74.6%) was considerably higher than the actual knowledge score (58.3%). Therefore, the schoolteachers, probably due to their social role as mentors for their students, are inclined to overestimate their oral health knowledge.

The knowledge domain of “dental trauma management” received the lowest score in both perceived knowledge and actual knowledge compared to other knowledge domains, thus suggesting that this is the most critical knowledge area that requires urgent educational interventions for schoolteachers in Turkey. A recent Turkish study found that pre-schoolteachers had inadequate knowledge about dental trauma management that might be attributed to lack of trauma experience of the teachers and suggesting the same call for educational interventions for both schoolteachers and parents on how to handle dental trauma, which is common in these age groups [51].

In Brazil, physical education undergraduates showed extremely low levels of knowledge about dental trauma [52]. The same was found in Hong Kong and India among physical education teachers [53,54]. In our sample, physical education teachers had the lowest frequency of correct answers to the actual knowledge questions of dental trauma management compared to teachers of other subjects. This finding supports the abovementioned call for educational interventions, especially for physical education teachers who are at the greatest risk of encountering dental fractures and avulsion events as part of their daily work with schoolchildren.

In our study, the schoolteachers with higher levels of perceived oral health knowledge had significantly higher levels of positive attitude towards oral health education. Ramroop et al., 2011 found that primary schoolteachers in Trinidad had satisfactory knowledge about aetiology and the prevention of dental caries, while they had inadequate knowledge about dental trauma management. In spite of that, Trinidadian teachers had positive attitudes towards oral health education, suggesting that actual knowledge may not necessarily predict teachers’ attitudes [55]. In Greece, India, and Nigeria, the schoolteachers’ inadequate knowledge about oral health was significantly associated with their willingness to learn about oral health and involved themselves in its teaching [56,57,58].

However, the overall oral hygiene behaviour score was weakly correlated with the overall score of perceived knowledge and actual knowledge; teachers’ hygiene behaviours were not found to be associated with positive attitudes towards oral health education, nor were teachers’ experience with teaching about oral health. Similarly, in Saudi Arabia, the high levels of oral hygiene were not associated with positive attitudes towards oral health education among schoolteachers [39]. These findings contrast with what was reported earlier by Maganur et al., 2017 in India and Dawani et al., 2013 in Pakistan, where schoolteachers with good oral hygiene had a significantly higher level of knowledge and more positive attitudes towards oral health education [59,60]. Our findings can be either explained as a methodological limitation because the oral hygiene items adapted from HU-DBI may not have been supposed to be taken out of their context, or as a true phenomenon where personal oral hygiene of the teachers’ may not affect their attitudes and practice of oral health education. This point warrants further investigation to understand better the role of schoolteachers’ oral hygiene in shaping their students’ oral health behaviours and attitudes.

Our schoolteachers’ perceived oral health knowledge, actual knowledge and attitudes were significantly associated with their experience of providing oral health education to their students. This finding highlights the importance of increasing teachers’ awareness through educational interventions as a strategy to increase their likelihood of providing actual guidance and education to their students.

### 4.1. Study Limitations

The first limitation of this study is its sample, which was not equally stratified across gender, but this can be justified as the sample was aimed to be as representative as possible for the actual demographics of schoolteachers in Turkey who are predominantly female. The second limitation is that the vast majority of respondents were from public schools; however, the number of public schools is very close to the number of private schools in Istanbul. The third limitation was the selection bias, which was unavoidable in the convenience (non-random) sampling approach that we followed in this study; the teachers who voluntarily joined the study may have better awareness regarding oral health and oral hygiene issues.

### 4.2. Study Strengths

To the best of the authors’ knowledge, this is the first study to evaluate schoolteachers’ oral health knowledge, attitudes, and behaviours in the largest Turkish metropolis (Istanbul). It is the first study, to date, comparing teachers’ perceived knowledge and actual knowledge aiming to explore knowledge discrepancies of the target population. The study provides support to the prevailing evidence on the role of oral health knowledge in shaping teachers’ attitudes towards oral health education, their oral hygiene behaviours, and their experience with teaching their students about oral health.

### 4.3. Study Implications

The findings of this study suggest that future studies of schoolteachers’ oral health knowledge should discriminate clearly between perceived knowledge and actual knowledge, and they should rely entirely on perceived knowledge outcomes as they can be overestimated. The schoolteachers, especially physical education teachers, exhibited low levels of knowledge about dental trauma management, thus calling for urgent educational intervention to increase their awareness and skills. A conceptual model for oral health knowledge, attitudes and practice of elementary schoolteachers should be proposed and tested in the future based on our study data and other similar studies which employed various oral health constructs, e.g., knowledge, attitudes, behaviours, etc. This suggested model will serve as a basis for promotion interventions targeting schoolchildren and their teachers.

## 5. Conclusions

The elementary schoolteachers in Istanbul, Turkey, showed satisfactory oral health knowledge and attitudes toward oral health education. The correlation between their perceived knowledge and actual knowledge was very weak, suggesting that the teachers are inclined to overestimate their knowledge. The teachers’ knowledge about dental trauma management was inadequate, necessitating urgent educational interventions, especially for physical education teachers, who are at the greatest risk of encountering such events during their work. Female gender and greater work experience were found to be promoters for oral health knowledge and positive attitudes. The oral hygiene behaviours were not clearly associated with teachers’ oral health knowledge, attitudes or practice, thus requiring further investigation.

## Figures and Tables

**Table 1:** Demographic characteristics of elementary schoolteachers, Istanbul, Turkey, 2020.

Variable	Outcome	Frequency	Percentage	Cumulative Percentage
Gender	Female	241	62.2%	62.2%
	Male	144	37.4%	100%
Experience	1–5 years	87	22.6%	22.6%
	6–10 years	111	28.8%	51.4%
	11–20 years	89	23.1%	74.5%
	>20 years	98	25.5%	100%
Qualification	License	313	81.3%	81.3%
	Masters	66	17.1%	98.4%
	Other	6	1.6%	100%
School Type	Public	333	86.5%	86.5%
	Private	52	13.5%	100%
Branch	Physical Education	11	2.9%	2.9%
	Other	312	97.1%	100%

**Table 2:** Oral health-related knowledge of elementary schoolteachers stratified by work experience, Istanbul, Turkey, 2020.

Item	Outcome	≤10 Years	>10 Years	Total	Significance
**Perceived Knowledge**					
I am aware enough of primary teeth	Strongly Agree	38 (19.2%)	62 (33.2%)	100 (26%)	
	Agree	110 (55.6%)	85 (45.5%)	195 (50.6%)	
	Not Sure	39 (19.7%)	35 (18.7%)	74 (19.2%)	
	Disagree	11 (5.6%)	3 (1.6%)	14 (3.6%)	
	Strongly Disagree	0 (0%)	2 (1.1%)	2 (0.5%)	
	(1–5)	3.88 ± 0.78	4.08 ± 0.82	3.98 ± 0.8	0.010
I am aware enough of oral diseases etiologies	Strongly Agree	26 (13.1%)	44 (23.5%)	70 (18.2%)	
	Agree	85 (42.9%)	92 (49.2%)	177 (46%)	
	Not Sure	67 (33.8%)	38 (20.3%)	105 (27.3%)	
	Disagree	17 (8.6%)	10 (5.3%)	27 (7%)	
	Strongly Disagree	3 (1.5%)	3 (1.6%)	6 (1.6%)	
	(1–5)	3.58 ± 0.88	3.88 ± 0.89	3.72 ± 0.9	<0.001
I am aware enough of oral hygiene methods	Strongly Agree	38 (19.2%)	49 (26.2%)	87 (22.6%)	
	Agree	129 (65.2%)	101 (54%)	230 (59.7%)	
	Not Sure	22(11.1%)	29 (15.5%)	51 (13.2%)	
	Disagree	7 (3.5%)	6 (3.2%)	13 (3.4%)	
	Strongly Disagree	2 (1%)	2 (1.1%)	4 (1%)	
	(1–5)	3.98 ± 0.73	4.01 ± 0.8	3.99 ± 0.77	0.589
I am aware enough of what to do in case of tooth injury (dental trauma)	Strongly Agree	11 (5.6%)	20 (10.7%)	31 (8.1%)	
	Agree	48 (24.2%)	63 (33.7%)	111 (28.8%)	
	Not Sure	88 (44.4%)	81 (43.%)	169 (43.9%)	
	Disagree	41 (20.7%)	17 (9.1%)	58 (15.1%)	
	Strongly Disagree	10 (5.1%)	6 (3.2%)	16 (4.2%)	
	(1–5)	3.05 ± 0.94	3.40 ± 0.91	3.22 ± 0.94	<0.001
**Actual Knowledge**					
Number of primary teeth	True	89 (44.9%)	72 (38.5%)	161 (41.8%)	0.200
First permanent tooth	True	95 (48%)	71 (38%)	166 (43.1%)	0.047
Caries of primary teeth	True	103 (52%)	114 (61%)	217 (56.4%)	0.077
Sweet snacks	True	177 (89.4%)	163 (87.2%)	340 (88.3%)	0.496
Acidic food	True	180 (90.9%)	171 (91.4%)	351 (91.2%)	0.853
Thumb sucking	True	141 (71.2%)	145 (77.5%)	286 (74.3%)	0.156
Dental attendance frequency	True	149 (75.3%)	134 (71.7%)	283 (73.5%)	0.424
Oral hygiene aids	True	118 (59.6%)	98 (52.4%)	216 (56.1%)	0.155
Avulsed tooth medium	True	41 (20.7%)	59 (31.6%)	100 (26%)	0.015
Avulsed tooth replantation	True	55 (27.8%)	70 (37.4%)	125 (32.5%)	0.043
Knowledge Domain No. 1	(0–2)	0.93 ± 0.8	0.76 ± 0.75	0.85 ± 0.78	0.043
Knowledge Domain No. 2	(0–4)	3.04 ± 1.15	3.17 ± 1.16	3.1 ± 1.15	0.084
Knowledge Domain No. 3	(0–2)	1.35 ± 0.71	1.24 ± 0.67	1.3 ± 0.69	0.081
Knowledge Domain No. 4	(0–2)	0.48 ± 0.72	0.69 ± 0.8	0.58 ± 0.77	0.007
Total	(0–10)	5.8 ± 1.63	5.87 ± 1.82	5.83 ± 1.76	0.651

Chi-squared test (*χ*^2^) and Mann–Whitney (*U*) test were used with a significance level of ≤0.05.

**Table 3:** Oral health-related behaviours of elementary schoolteachers stratified by work experience.

Item	Outcome	≤10 Years	>10 Years	Total	Significance
I have noticed white stick deposits on my teeth	Disagree	98 (49.5%)	63 (33.7%)	161 (41.8%)	0.002
I use a child-sized toothbrush	Agree	20 (10.1%)	30 (16%)	50 (13%)	0.083
I have used a dye to see how clean my teeth are	Agree	8 (4%)	21 (11.2%)	29 (7.5%)	0.008
I put off going to the dentist until I have toothache	Disagree	106 (53.5%)	97 (51.9%)	203 (52.7%)	0.744
I do not worry about visiting the dentist	Agree	117 (59.1%)	126 (67.4%)	243 (63.1%)	0.092
Total	(0–5)	1.76 ± 0.95	1.8 ± 0.95	1.78 ± 0.95	0.739

Chi-squared test (*χ*^2^) and Mann–Whitney (*U*) test were used with a significance level of ≤0.05.

**Table 4:** Oral health-related attitudes of elementary schoolteachers stratified by work experience.

Item	Outcome	≤10 Years	>10 Years	Total	Significance
Do you think that teachers should have a role in oral health education of schoolchildren?	Strongly Agree	52 (26.3%)	56 (29.9%)	108 (28.1%)	
	Agree	95 (48%)	108 (57.8%)	203 (52.7%)	
	Not Sure	30 (15.2%)	15 (8%)	45 (11.7%)	
	Disagree	19 (9.6%)	6 (3.2%)	25 (6.5%)	
	Strongly Disagree	2 (1%)	2 (1.1%)	4 (1%)	
	(1–5)	3.89 ± 0.94	4.12 ± 0.77	4 ± 0.87	0.017
Do you think teachers should receive oral health training as a part of general health training?	Strongly Agree	44 (22.2%)	48 (25.7%)	92 (23.9%)	
	Agree	96 (48.5%)	106 (56.7%)	202 (52.5%)	
	Not Sure	38 (19.2%)	17 (9.1%)	55 (14.3%)	
	Disagree	16 (8.1%)	11 (5.9%)	27 (7%)	
	Strongly Disagree	4 (2%)	5 (2.7%)	9 (2.3%)	
	(1–5)	3.81 ± 0.94	3.97 ± 0.91	3.89 ± 0.93	0.048
Do you think oral health can affect the psychology of schoolchildren?	Strongly Agree	52 (26.3%)	51 (27.3%)	103 (26.8%)	
	Agree	114 (57.6%)	117 (62.6%)	231 (60%)	
	Not Sure	20 (10.1%)	13 (7%)	33 (8.6%)	
	Disagree	10 (5.1%)	3 (%1.6)	13 (3.4%)	
	Strongly Disagree	2 (1%)	3 (1.6%)	5 (1.3%)	
	(1–5)	4.03 ± 0.81	4.12 ± 0.73	4.08 ± 0.76	0.303
Do you think oral health education can benefit your schoolchildren?	Strongly Agree	65 (32.8%)	76 (40.9%)	141 (36.7%)	
	Agree	112 (56.6%)	98 (52.7%)	210 (54.7%)	
	Not Sure	15 (7.6%)	8 (4.3%)	23 (6%)	
	Disagree	4 (2%)	1 (0.5%)	5 (1.3%)	
	Strongly Disagree	2 (1%)	3 (1.6%)	5 (1.3%)	
	(1–5)	4.18 ± 0.74	4.31 ± 0.73	4.24 ± 0.73	0.058
Total	(5–20)	15.91 ± 2.83	16.54 ± 2.48	16.21 ± 2.68	0.019

Mann-Whitney (*U*) test was used with a significance level of ≤0.05.

**Table 5:** Oral health education experience of elementary schoolteachers stratified by work experience.

Item	Outcome	≤10 Years	>10 Years	Total	Significance
Education of general hygiene	True	24 (12.1%)	43 (23%)	67 (17.4%)	0.005
Education of oral hygiene	True	14 (7.1%)	22 (11.8%)	36 (9.4%)	0.114
Checking children’s teeth	True	10 (5.1%)	19 (10.2%)	29 (7.5%)	0.058
Supervising children’s brushing	True	102 (51.5%)	97 (51.9%)	199 (51.7%)	0.944
Teaching oral health	True	51 (25.8%)	59 (31.6%)	110 (28.6%)	0.209
Total	(0–5)	1.02 ± 1.1	1.28 ± 1.27	1.15 ± 1.19	0.044
Teaching oral health: topics	Dental Anatomy	4 (8%)	6 (11.5%)	10 (9.8%)	0.476
	Oral Hygiene	42 (84%)	43 (82.7%)	85 (83.3%)	
	Oral Diseases	4 (8%)	3 (5.8%)	7 (6.9%)	
Teaching oral health: methods	Oral Health Videos	27 (52.9%)	41 (78.8%)	68 (66%)	0.005
	Mouth Models	17 (33.3%)	9 (17.3%)	26 (25.2%)	
	Printed Posters	7 (13.7%)	2 (3.8%)	9 (8.7%)	
Teaching oral health: feedback	Very Favorable	14 (27.5%)	14 (26.4%)	28 (26.9%)	0.902
	Favorable	25 (49%)	26 (49.1%)	51 (49%)	
	Not Sure	9 (17.6%)	10 (18.9%)	19 (18.3%)	
	Unfavorable	2 (3.9%)	3 (5.7%)	5 (4.8%)	
	Very Unfavorable	1 (2%)	0 (0%)	1 (1%)	

Chi-squared test (*χ*^2^) and Mann–Whitney (*U*) test were used with a significance level of ≤0.05.

**Table 6:** Social determinants of elementary schoolteachers’ oral health knowledge, behaviours, and attitudes.

Variable	Outcome	Perceived Knowledge		Actual Knowledge		Behaviours		Attitudes	
		Mean ± SD	Significance	Mean ± SD	Significance	Mean ± SD	Significance	Mean ± SD	Significance
Gender	Female	15.16 ± 2.73	0.003	6.01 ± 1.76	0.011	1.83 ± 0.99	0.175	16.19 ± 2.78	0.861
	Male	14.49 ± 2.54		5.53 ± 1.63		1.70 ± 0.87		16.26 ± 2.51	
Experience	≤10 years	14.48 ± 2.57	0.001	5.80 ± 1.63	0.651	1.76 ± 0.95	0.739	15.91 ± 2.83	0.019
	>10 years	15.36 ± 2.71		5.87 ± 1.82		1.80 ± 0.95		16.54 ± 2.48	
Qualification	License	14.87 ± 2.66	0.154	5.77 ± 1.71	0.365	1.73 ± 0.93	0.006	16.14 ± 2.67	0.306
	Masters	14.95 ± 2.83		6.14 ± 1.83		2.09 ± 0.97		16.53 ± 2.77	
	Other	16.67 ± 1.37		5.83 ± 0.75		1.33 ± 1.03		16.67 ± 2.58	
School Type	Public	14.85 ± 2.76	0.312	5.85 ± 1.73	0.544	1.80 ± 0.93	0.222	16.20 ± 2.69	0.871
	Private	15.31 ± 2.06		5.71 ± 1.68		1.65 ± 0.99		16.31 ± 2.62	
Branch	Physical Education	13.18 ± 2.71	0.027	4.27 ± 1.95	0.005	1.73 ± 1.10	0.956	15.10 ± 3.11	0.107
	Other	14.96 ± 2.66		5.88 ± 1.70		1.78 ± 0.95		16.24 ± 2.67	

Mann–Whitney (*U*) and Kruskal–Wallis (*H*) test were used with a significance level of ≤0.05.

**Table 7:** Correlation of perceived oral health knowledge and actual knowledge of elementary schoolteachers.

Variable		Act. Knowledge I	Act. Knowledge II	Act. Knowledge III	Act. Knowledge IV	Act. Knowledge Total
Prc. Knowledge I	*ρ*.	0.080	0.203 **	0.080	0.196 **	0.264 **
	Sig.	0.117	<0.001	0.115	<0.001	<0.001
Prc. Knowledge II	*ρ.*	0.130 *	0.208 **	0.025	0.172 **	0.256 **
	Sig.	0.011	<0.001	0.629	0.001	<0.001
Prc. Knowledge III	*ρ.*	0.046	0.121*	0.071	0.140 *	0.169 **
	Sig.	0.369	0.017	0.164	0.006	0.001
Prc. Knowledge IV	*ρ.*	0.078	0.047	0.008	0.274 **	0.116 *
	Sig.	0.127	0.355	0.880	<0.001	0.023
Prc. Knowledge Total	*ρ.*	0.130 *	0.177 **	0.058	0.257 **	0.265 **
	Sig.	0.010	<0.001	0.254	<0.001	<0.001

Prc. = perceived knowledge; Act. = actual knowledge; *ρ*. = Spearman’s correlation coefficient; ** correlation is significant at the level 0.01 level (two-tailed); * correlation is significant at the 0.05 level (two-tailed). Sig. = Significance

**Table 8:** Impact of elementary schoolteachers’ oral health knowledge on their behaviours.

Variable	Outcome	Perceived Knowledge		Actual Knowledge	
		Mean ± SD	Significance	Mean ± SD	Significance
I have noticed white stick deposits on my teeth	Agree	15.10 ± 2.86	0.019	5.58 ± 1.61	0.006
	Disagree	14.65 ± 2.38		6.17 ± 1.83	
I use a child-sized toothbrush	Disagree	14.81 ± 2.67	0.052	5.84 ± 1.73	0.745
	Agree	15.60 ± 2.64		5.76 ± 1.71	
I have used a dye to see how clean my teeth are	Disagree	14.91 ± 2.60	0.644	5.83 ± 1.73	0.916
	Agree	14.93 ± 3.58		5.83 ± 1.71	
I put off going to the dentist until I have toothache	Agree	14.35 ± 2.50	<0.001	5.65 ± 1.64	0.130
	Disagree	15.41 ± 2.74		6.00 ± 1.79	
I do not worry about visiting the dentist	Disagree	14.66 ± 2.72	0.121	5.63 ± 1.77	0.094
	Agree	15.06 ± 2.65		5.95 ± 1.69	
Total Behavior	*ρ.*	0.138		0.146	
	Sig.	0.007		0.004	

Mann–Whitney (*U*) test and Spearman’s correlation (*ρ*) test were used with a significance level of ≤0.05.

**Table 9:** Impact of elementary schoolteachers’ oral health knowledge on their attitudes.

Variable	Outcome	Perceived Knowledge		Actual Knowledge	
		Mean ± SD	Significance	Mean ± SD	Significance
Do you think that teachers should have a role in oral health education of schoolchildren?	Disagreement	13.10 ± 3.30	0.002	5.24 ± 1.66	0.058
	Agreement	15.25 ± 2.60		5.97 ± 1.72	
Do you think teachers should receive oral health training as a part of general health training?	Disagreement	13.44 ± 3.30	0.001	5.19 ± 1.65	0.016
	Agreement	15.33 ± 2.51		6.02 ± 1.73	
Do you think oral health can affect the psychology of schoolchildren?	Disagreement	12.89 ± 4.03	0.041	5.94 ± 1.39	0.782
	Agreement	15.15 ± 2.49		5.88 ± 1.74	
Do you think oral health education can benefit your schoolchildren?	Disagreement	10.70 ± 4.00	0.001	5.30 ± 0.95	0.267
	Agreement	15.14 ± 2.52		5.90 ± 1.72	

Mann–Whitney (*U*) test was used with a significance level of ≤0.05; Disagreement = Totally Disagree + Disagree; Agreement = Totally Agree + Agree.

**Table 10:** Impact of elementary schoolteachers’ oral health knowledge and attitudes on their practice.

Variable	Outcome	Perceived Knowledge		Actual Knowledge		Attitudes	
		Mean ± SD	Significance	Mean ± SD	Significance	Mean ± SD	Significance
Education of general hygiene	False	14.59 ± 2.64	<0.001	5.72 ± 1.68	0.005	16.00 ± 2.71	<0.001
	True	16.43 ± 2.31		6.34 ± 1.85		17.24 ± 2.30	
Education of oral hygiene	False	14.78 ± 2.62	0.001	5.74 ± 1.67	0.008	16.06 ± 2.72	<0.001
	True	16.19 ± 2.93		6.69 ± 2.05		17.72 ± 1.67	
Checking children’s teeth	False	14.85 ± 2.58	0.043	5.79 ± 1.71	0.252	16.11 ± 2.68	0.005
	True	15.72 ± 3.63		6.28 ± 1.83		17.52 ± 2.36	
Supervising children’s brushing	False	14.80 ± 2.67	0.485	5.36 ± 1.60	<0.001	15.83 ± 2.80	0.006
	True	15.02 ± 2.69		6.27 ± 1.73		16.57 ± 2.52	
Teaching oral health	False	14.66 ± 2.61	0.002	5.64 ± 1.59	0.002	15.96 ± 2.66	0.001
	True	15.55 ± 2.75		6.32 ± 1.95		16.85 ± 2.63	

Mann–Whitney test was used with a significance level of ≤0.05.

## Data Availability

The data that support the findings of this study are available from the corresponding author upon reasonable request.

## Supplementary Materials

The following are available online at https://www.mdpi.com/article/10.3390/ijerph18116028/s1, Questionnaire of Schoolteachers’ Oral Health KAB (in Turkish).
