# Supplementary material for: Oral Health-Related Knowledge, Attitudes and Behaviours of Elementary School Teachers
Source: Int J Environ Res Public Health. 2021 Jun 3;18(11):6028. doi: 10.3390/ijerph18116028 (PMC8199972; doi:10.3390/ijerph18116028)
Supplement: Supplementary file 1 [file ijerph-18-06028-s001.zip › School Teachers Oral Health KAP Questionnaire.pdf]

[Questionnaire] Elementary School Teachers' Oral Health Knowledge, Attitudes and Behaviors

I. **Personal Information** (5 Questions)

1. Gender

   □ Female

   □ Male

2. Experience

   □ 1-5 years

   □ 6-10 years

   □ 11-20 years

   □ +21 years

3. Education Status

   □ B.Ed.

   □ M.Ed.

   □ Others

4. School

   □ ………..

5. Branch

   □ Physical Education

   □ Mathematics

   □ Literature

   □ Other

II. **Perceived Knowledge** (4 Questions)

1. I am aware enough of primary teeth

   □ Strongly Disagree □ Disagree □ Not Sure □ Agree □ Strongly Agree

2. I am aware enough of oral diseases origins

□ Strongly Disagree □ Disagree □ Not Sure □ Agree □ Strongly Agree

3. I am aware enough of oral hygiene techniques

□ Strongly Disagree □ Disagree □ Not Sure □ Agree □ Strongly Agree

4. I am aware enough of what to do in case of tooth injury (dental trauma)

□ Strongly Disagree □ Disagree □ Not Sure □ Agree □ Strongly Agree

III. **Actual Knowledge** (10 Questions)

1. There are …… primary (milky) teeth in total.

□ 16 □ 20 □ 24 □ I do not know

2. The first permanent (adult) tooth appears at the age of …... years old.

□ 5 □ 6 □ 7 □ I do not know

3. Caries of primary (milky) teeth can affect the permanent (adult) teeth.

□ True □ False □ I do not know

4. Sweet snacks (wafer biscuits and cake) can cause tooth decay.

□ True □ False □ I do not know

5. Acidic food can cause tooth decay.

□ True □ False □ I do not know

6. Thumb sucking may cause dentofacial deformities.

□ True □ False □ I do not know

7. To prevent tooth decays, the average child should visit the dentist …......

□ every 3 months □ every 6 months □ every 1 year □ I do not know

8. To prevent tooth decays, toothbrushing should be followed by ……...

□ mouth rinsing □ flossing □ both □ I do not know

9. In case of dental trauma, the avulsed tooth should be put in ……

□ saline □ tape-water □ milk □ I do not know

10. The avulsed tooth can be replanted …….

□ True □ False □ I do not know

IV. Attitudes (3 Questions)

1. Do you think that teachers should have a role in oral health education of schoolchildren?

□ Strongly Disagree □ Disagree □ Not Sure □ Agree □ Strongly Agree

2. Do you think teachers should receive oral health training as a part of general health training?

□ Strongly Disagree □ Disagree □ Not Sure □ Agree □ Strongly Agree

3. Do you think oral health can affect the psychology of schoolchildren?

□ Strongly Disagree □ Disagree □ Not Sure □ Agree □ Strongly Agree

4. Do you think oral health education can benefit your schoolchildren?

□ Strongly Disagree □ Disagree □ Not Sure □ Agree □ Strongly Agree

V. Behaviors (5 Questions)

1. I have noticed some white stick deposits on my teeth

□ Agree □ Disagree

2. I use a child sized toothbrush.

□ Agree □ Disagree

3. I have used a dye to see how clean my teeth are.

□ Agree □ Disagree

4. I put off going to the dentist until I have a toothache.

□ Agree □ Disagree

5. I don't worry about visiting the dentist

□ Agree □ Disagree

VI. Practice (8 Questions)

1. Have you been trained to provide education on topics related to general health and hygiene?

□ True □ False

2. Have you been trained to provide education on topics related to oral hygiene?

□ True □ False

3. I elevate the upper lip of my schoolchildren and check their teeth.

□ True □ False

4. I supervise the brushing habit of my schoolchildren.

□ True □ False

5. Have you tried to provide education on topics related to teeth and oral health?

□ True □ False

6. If the answer of Q5 is Yes, what kind of oral health education have you tried to provide to your schoolchildren? (select all that apply)

□ Teeth types, function, structure, and eruption

□ Toothbrushing, healthy nutrition, risky oral habits

□ Tooth decay, gum diseases, and misarranged (irregular) teeth

7. If the answer of Q5 is Yes, what methods have you used in providing oral health education to your schoolchildren? (select all that apply)

□ Oral Health Videos

□ Mouth Model

□ Printed Posters

8. If the answer of Q5 is Yes, how do you evaluate your students' response?

□ Very Favorable □ Favorable □ Not sure □ Unfavorable □ Very Unfavorable
